# Determinants of sickness absence in police: Case study of Abu Dhabi police department, UAE

**DOI:** 10.1016/j.heliyon.2023.e23714

**Published:** 2023-12-15

**Authors:** Faisal Almurbahani Alkaabi, Praveen Kumar Maghelal, Jana Ahmed AlShkeili

**Affiliations:** a Faculty of Resilience, Rabdan Academy, United Arab Emirates; b Rabdan Academy, United Arab Emirates

**Keywords:** Sickness absence, Police, Abu Dhabi, United Arab Emirates, Occupational health

## Abstract

Sickness absence among employees is reported to reduce organization profits and performance and thus threaten the organization's existence in the market. The monitoring and reporting of data on sickness absence is considered a crucial element of reactive health and safety control systems in organizations. It is one of the major indicators of organizational continuous commitment to improving the quality of working conditions. However, sickness absence in Police in the United Arab Emirates (UAE) is less investigated. The Occupational Health and Safety Survey developed for this study was distributed to 1317 employees of the Capital Police Directorate of Abu Dhabi Police. The survey was answered by 760 employees (58 %). While 230 (17 %) refused to participate, 259 (20 %) did not return the surveys, and 68 (5 %) were not surveyed as they were on authorized long-term leave for various reasons (and did not receive the survey). This study analyzes if the psychosocial work factors, physical work exposure factors, and employee's perception of the health and safety management system predict sickness absence in the Abu Dhabi Police after taking into account the other covariates. This study found no association between job control and the risk of sickness absence, in contrast to findings from other studies. There was also no association between psychological job demand and the perception of health and safety management with the risk of sickness absence in this study. Officers who fit the ‘job strain’ category did not have a significant increase in the risk of sickness absence in this study. However, high levels of combined physical exposures reported a significant relation with sickness absence. In conclusion, this, being one of the first studies in the region, provides insights on work factors and perception of HSE on sickness absence and provides recommendations within the context of the region for future studies and address sickness absence among police in the UAE.

## 1 Introduction

The monitoring of sickness absence is considered a crucial element of organizational reactive health and safety control systems. It is one of the major indicators of an organization's commitment to improving the quality of working conditions [1]. The term ‘sickness absence’ refers to any absence from work that is attributed to sickness by the employee and accepted as such by the employer [2]. At an individual level, sickness absence is a method of communicating illnesses and is influenced by work and non-work factors. It is associated with morbidity including disability [leading to disability retirement], mortality [3], isolation and inactivity [4,5], social problems [6] and exclusion from the labour market [7]. From an occupational health perspective, sickness absence could be considered as an indicator of job characteristics such as complexity of tasks, work environment, exposure to substances, ergonomics, and other factors [8,9]. Thus, sickness absence may provide evidence of the quality of health and safety management systems provided by the organization [1].

The health and safety management system, first prepared in the UK in 1991, is a practical guide for an organization's directors, managers, employees, and specifically health and safety professionals [10]. This guide includes a set of policies, procedures, and plans to monitor the health and safety in an organization to mitigate the risk of injuries from everyday operations systematically. This organization-wide approach not only accumulates the health and safety data of its employees but also uses feedback to better manage and improve the health and safety of its employees [11].

The monitoring and reporting of data on sickness absence is essential for several reasons. First, the data collected can be used to provide indirect measurements of the effectiveness of risk control systems and workplace exposure. Monitoring such occupational data is considered both an active and a reactive monitoring system [1]. Monitoring of occupational health data may also provide more insights into the mediating mechanisms and causal processes of the relationship between work characteristics and employees’ health. This, in turn, will facilitate a better design of the health and safety systems at the workplace [12].

Monitoring may also help to reduce sickness absence, which in turn will minimise financial loss both at an organizational or governmental level occurring through the loss of productivity. Organizations may struggle to replace competent and knowledgeable staff for longer periods, while costs increase at the governmental level owing to increasing demands on government pensions and health care systems [1,13]. Furthermore, monitoring employees' health is crucial as employees suffering from occupational stress, for example, tend to be less motivated, adopt unhealthy and unsafe behaviours and be less productive which may reduce organizational profits and performance and thus, threaten the organisation's existence in the market Leka et al. [14].

Conversely, due to the complexity of their operational activities and multicultural working environment, the rate of sickness absences and occupational injuries in organization such as the police, fire fighting and other emergency response services is higher than in other organization [15]. Police work involves exposure to certain occupational hazards that might endanger the health of officers, such as handling law enforcement, exposure to violent situations, negative attitudes or even threats from the public, and uncomfortable work schedules. Thus, due to the complexity of their operational activities and working environment, the rate of sickness absences and occupational injuries in organization such as the police, fire fighting and other emergency response services is higher than in other organization [[15], [16], [17]].

The literature identifies two broad sets of determinants of sickness absence, namely, non-work-related factors and work-related factors. While non-work-related factors have been investigated extensively over the last two decades and report a somewhat consistent relationship with sickness absence, work-related factor results have been inconsistent, and its impact on sickness absence in police for the region (UAE) has never been investigated. The Job Demand-Control model developed by Karasek [18], involving the two dimensions of work-related (demand) and non-work-related (control) factors, has been validated and used extensively to analyze sickness absence in many countries. While the non-work-related factors have mostly reported consistent relationships, the work-related factors report varying relations with sickness absence. Henceforth, this study focuses on the impact of work-related factors on sickness absence in the UAE police force, adjusting for gender and age. This study uses a cross-section survey design to analyze if the psychosocial, physical, and employee perceptions of the health and safety management system predict sickness absence in the Abu Dhabi Police after taking into account other covariates such as gender and age.

While studies have analyzed the determinant of disability retirement in Abu Dhabi Police [19], the evaluation of sickness absence in the Police force, one of UAE's largest employee recruiters, is lacking. Sickness absence is an important source of productivity loss and may also serve as an indirect measure of the police force's morbidity [20]. In some cases, the absence has increased significantly, with the recurrence of such leave increasing early retirement intentions [21]. However, there is no understanding of what determines the sickness absence in the Abu Dhabi Police Department. Understanding this would provide the government with insights into specific policing occupational health and safety requirements and what could be done to manage their long-term health and safety better.

This is also critical to mitigate the rate of sickness leave in the future, not just in the emirate of Abu Dhabi but across all the seven emirates and the Gulf Cooperation Council nations. This study, therefore, aims to examine the role of psychosocial factors, physical exposure (at work), and perception of the health and safety management system of sickness absence of police employees of Abu Dhabi, UAE. It has to be noted that this is the first study that investigates the determinants of sickness absence for police employees in the UAE, thus providing a unique contribution in the sense of regional perspectives. Meeting these specific standards will enhance the productivity of officers, which, in turn, will maximize security and stability in the country.

## 2 Literature review

Police officers have a high risk of cardiovascular diseases [22], suicide [23] and occupational injuries [24]. For example, in 2012, the rate of injuries in the UK police force was four times higher than the rate for ‘all occupations.’ Similarly, higher rates for police were reported for stress, anxiety, and depression [24,25]. Although police jobs are intrinsically hazardous, studies have shown that policing tasks are mostly light physically, but officers are more likely to be involved in stressful situations [mentally demanding] that increase the risk of occupational stress [26]. Occupational stress is associated with sickness absence [27,28] and early medical retirement [15]. For some officers, particularly those who are not involved in fieldwork, the majority of working time is spent on sedentary tasks [29], but officers are required to show flexibility and adaptability to respond quickly to sudden changes in the working environment [30].

Thus, police departments face difficulties in meeting their legal health and safety obligations as occupational risks are inevitable; hence, they are encouraged to integrate their health and safety management systems with the operational arrangements. In the UK, the Health and Safety Executive [30], in co-operation with the UK Police force, published a document titled ‘Striking the balance between operational and health and safety duties in the Police,’ which recognized the difficulties in implementing health and safety regulations in police organization and provided recommendations to overcome such issues. One way to demonstrate commitment to the improvement of the working environment in the police is by the collection and analysis of occupational health data such as sickness absence and early retirement intention. A thorough analysis of such data will help police organization to prioritise plans and improve the health and well-being of officers, in turn contributing to strengthening the fight against crime and minimizing the number of occupational injuries and premature exit from paid work. The literature reviews the two major groups of determinants of sickness absence: the non-work and work-related factors.

### 2.1 Non-work related factors

These factors include age, gender, family factors, socioeconomic status (SES), lifestyle factors, health-related factors, and other factors. Literature generally shows that the risk of sickness absence increases in females [27,31], unmarried, divorced, separated and widows/widowers [32,33], individuals with low socio-economic status [SES] [33,34]. However, the association between age and sickness absence is mixed [35].

Regarding age, Svedberg & Alexanderson [36] showed that, compared with police officers who are 30 years of age or younger and after adjusting for gender, all other groups had a statistically higher risk of sickness absence with the highest increase in those between 40 and 55 followed by officers between 31 and 39 years old and officers who are 56 and above.

When stratified by gender, the risk remained statistically significant in females in all age groups (using the same reference group), and the risk increased up to 90 % (40–55 years old), 80 % (31–39 years old) and 56 % (≥56 years old). In males, the risk remained statistically significant only in officers between 41 and 55 years of age. Although some studies adjusted for age [[37], [38], [39]], they did not report age-related differences in risk of sickness absence. However, Boyce et al. [37] and Burke et al. [40] showed no significant differences in sickness absence rates between male and female police officers but Svedberg & Alexanderson [36] reported a significant 29 % increase in risk of sickness absence in female officers compared with male after adjusting for age, duration of employment, and type of employment.

As for the lifestyle and health-related factors, the risk of sickness absence also increases in current and former smokers [33,41,42], heavy, frequent and non-alcohol drinkers [the latter also known as ‘abstainers’] [43,44], problem drinkers [44], obese individuals [45] and physically inactive people [46,47]. A higher risk of sickness absence is also seen in individuals with the following; poor self-rated health [[48], [49]], health complaints [[28], [35], [50]], and low workability [51].

### 2.2 Work factors

The Job Demand-Control model to measure work-related stress was initially formulated by Karasek [18] and then developed both empirically and psychometrically by Karasek and Theorell in 1990 [52]. It is currently the most commonly used self-administered measure of occupational stress [53,54]. The model has two dimensions: the demand dimension and the control or decision latitude dimension. The demand dimension is concerned with the complexity of work tasks (their challenges and variety of tasks), their intensity, the skills demanded, and the possibility of keeping up with co-workers, while the control dimension is sub-divided into factors that measure decision authority (‘one's control over work situation’) and skill discretion (‘opportunities available to learn skills and competencies’) [18,52].

The dimensions of this model are ‘interactive’ [18,55] because, within this model, employees can be arranged into four work-status categories, namely, active (high control/high demand), passive (low control/low demand), relaxed (high control/low demand) and job strain (low control/high demand). As many studies have shown that reduced social support at work enhances the negative effects of high job strain [[56], [57], [58]], the model was extended to incorporate social support. This dimension included questions regarding support from both supervisors and colleagues.

Studies have shown that job strain is associated with many adverse health outcomes such as cardiovascular diseases [59], mental illnesses [52,60], functional decline in health status [61] and adverse reproductive outcomes [62]. Some studies, on the other hand, did not find an association [63,64].

There are two versions of JCQ – the full version consists of 49 items and the shorter version consists of 22 items. The shorter version of JCQ, which is most commonly used, contains nine decision latitude items (six items for skills discretion and three on decision authority), five psychological job demand items, and eight workplace support items (supervisors support (four items) and co-workers’ support (four items)).

The influence of work factors on sickness absence is evaluated using three broad sub-classifications described by Laaksonen et al. [65] and Lahelma et al. [66], namely, psychosocial working conditions (psychosocial exposure), physical working conditions (or physical exposure) and work arrangements.

#### 2.2.1 Psychosocial factors

The greater part of the literature investigating the influence of psychosocial work factors on sickness absence is based on the job-demand-control model of Karasek [18]. The influence of high demand on sickness absence is mixed [[27], [31]], while high job control is shown to reduce sickness absence [27,33]. Low control [[52], [67]] and high job demand [68,69] have been linked to psychological stress and many illnesses that are associated with the increased risk of sickness absence [70,71]. The combination of the two dimensions [high demand and low control], also known as ‘job strain’ has a stronger effect on health outcomes such as sickness absence than the total effect of the two dimensions considered separately [[18], [52]].

In general, higher support at work minimises sickness absences [[70], [72], [73]] while a few studies have found an association in only males [29] or females [74] or even a non-significant association [[27], [75]]. Social support has a buffering influence on stress, protecting workers from the pathological consequences of stressful experiences.

#### 2.2.2 Physical characteristics

The literature review conducted by Allebeck and Mastekaasa [76] on predictors of sickness absence indicated that evidence on the influence of physical work characteristics on sickness absence is ‘limited’, with poor physical work ergonomics being the strongest of all physical factors as a predictor of sickness absences. Adverse working conditions reduce job satisfaction [51] and cause job stress [77] which, in turn, is associated with many illnesses that increase sickness absenteeism [28,78].

#### 2.2.3 Work arrangements

Shift work, particularly the evening shift [79], increases sickness absence because it increases fatigue [80] and conflicts between work and family and is linked to many illnesses [81]. The risk of sickness absence may differ between various job types [[27], [33]]. This relationship is influenced mainly by other factors such as job characteristics [82], socioeconomic status [48], available support, and the representations of genders in different work areas [83]. English et al. [84] also found that police officers who were exposed to acutely toxic materials (the cases) did not have a significantly higher risk of sickness absence than those who were not exposed (the controls) from day 1–7 days after their exposure.

Other instances of exposure that have been linked to an increasing risk of sickness absence in the police significantly include driving a car for long periods as part of the job [85], police stress [86], career progression frustration (only in females), exposure to major manhunt [87] and passive smoking (only in males). Only one study investigated the influence of the job demand, control and support model as well as the effort/reward imbalance model [38] on number of days of sickness absence.

While several studies have investigated the role of psychosocial factors, the combined influence of physical working conditions (or physical exposure), work arrangements, and perception of health and safety management is mostly ignored. Moreover, such studies for the Middle Eastern region, specifically in the UAE, are lacking, given the inaccessibility of such data due to strict privacy concerns. Additionally, law enforcement agencies in the region have not been investigated for the impact of work-related factors on sickness absence.

Therefore, this study evaluates the influence of work factors, in particular psychosocial and physical factors and employees' perception of the health and safety management systems implemented by the organisation, on sickness absence in the Abu Dhabi Police. The specific objectives include [1] to evaluate the association and magnitude of the potential relationship between work factors, psychosocial, physical, and employees’ perceptions of health and safety dimensions as well as their overall perception of the health and safety management system on the risk of sickness absence, and [2] to investigate whether any observed associations between work factors and sickness absence are explained by other work and non-work factors.

## 3 Methodology

### 3.1 Study area

Abu Dhabi is the capital of the United Arab Emirates (UAE) and represents 87 % of its total area. Abu Dhabi sits on top of 10 % of the world's oil reserves and 5 % of global natural gas reserves. The Abu Dhabi government was successful in reducing economic dependency on oil in 2021. 49.7 % of its Gross Domestic Product (GDP) (417,735 million out of 840,513 million AED) was from non-oil economic activities [88]. Abu Dhabi citizens enjoy one of the highest standards of living worldwide as the per capita GDP increased from 305,500 AED (£67,226) in 2010 to 356,600 AED (£78,471) in 2019 [89].

The total population of Abu Dhabi in 2014 was 2.6 million, of whom approximately only one-fifth were UAE nationals (0.5 million). More than two-thirds (1.7 million) of the population were male, and 0.9 million were female. The majority of the population (82 %) were between 15 and 64 years of age, reflecting the expatriate immigration for work purposes in Abu Dhabi. In 2014, the number of physicians, nurses, and hospital beds per 1000 population was 2.8, 5.8, and 1.6 in the UAE [90] compared with 2.8, 9.5, and 3.0 in the UK [91]. Life expectancy at birth is 79.2 years and differs little between males and females and citizens and non-citizens. The distribution of the workforce by gender in Abu Dhabi in 2008 is shown in Table 1.

**Table 1:** Distribution of workforce in Abu Dhabi by gender.

Employment factor	UAE nationals		Total	Non-nationals		Total
	Males	Females	96,319	Males	Females	822,980
Labour force^a^	75,870	20,449		697,480	125,500	
Working population	68,074	18,198	86,272	685,558	119,587	803,145
Unemployment rate (%)	8.1	29.6	12.6	1.8	6.4	3.6

Data obtained from the Statistics Centre of Abu Dhabi [92].

a This includes people who are working and those who are looking for jobs.

### 3.2 Abu Dhabi police

Abu Dhabi Police is a part of the Ministry of Interior of the UAE and consists of six General Directorates at the time of this study. Each General Directorate consists of several Administrations, which are further divided into departments, branches, and units. The General Directorate of Policing Operations is the largest employee recruiter and the only one that has Directorates rather than Administrations within its organizational structure. Although official statistics are unpublished, Abu Dhabi Police employs over 35,000 police officers and civilians [90]. The police in the UAE primarily constitute UAE nationals, while non-nationals mainly work as civilians. The police occupation is also considered as one of the prestigious jobs in the UAE and officers are continuously encouraged to stay close to the public to gain more insights into their concerns to enhance public safety.

### 3.3 Survey design and data

Many studies have used Karasek's JCQ in different languages, including South Korean [93], Brazilian [94], Persian [95], and other languages. Thus, it was decided to use this measure to evaluate the influence of psychosocial work factors on sickness absence and early retirement intention in our police sample. However, as the JCQ had not yet been translated into Arabic, this study aimed to develop an Arabic version of this questionnaire [the Arabic JCQ] to survey the Abu Dhabi Police, one of the largest organizations in the Middle East.

An approval to distribute occupational health surveys to Abu Dhabi Police employees was granted by the Secretary General of the Office of His Highness Deputy Prime Minister and Minister of Interior of the 10.13039/100016565UAE in 2012. Ethical approval was also obtained from the Health Authority of Abu Dhabi (HAAD) in 2013. In 2014, an official letter was sent from the Human Resource Directorate of Abu Dhabi Police to the Capital Police Directorate to allow the distribution of the surveys to the employees of the latter Directorate. This Directorate was selected for two main reasons. First, a wide range of policing activities are carried out by this Directorate, including investigation, traffic, crime scene support, community policing, and other activities. Secondly, this Directorate has five police stations covering different areas of central Abu Dhabi with a total of 1425 employees. These stations are considered to be some of the busiest in fighting crime in the United Arab Emirates.

The Occupational Health and Safety Survey was distributed to 1317 employees of other departments of the Capital Police Directorate of Abu Dhabi Police between Feb 1st and March 31st^,^ 2015. The survey was answered by 760 employees (58 %); 230 (17 %) refused to participate; 259 (20 %) did not return the surveys; and 68 (5 %) were not surveyed as they were on an authorized long-term leave for various reasons (and did not receive the survey). Excluding that last group, the response rate was 61 % (760/1249). The survey contained a front page describing the objectives of the research along with the contact details of the researcher. After this section, participants were asked whether or not they consented to continue participating in the survey. All consented responses totaling 760 responses were used for further analysis. The study was performed in accordance with the relevant guidelines and regulations of the Declaration of Helsinki, and the survey responses were anonymized before its use for this study.

### 3.4 Demographic characteristics

Table 2 describes the demographic characteristics of the main study sample. The mean age of the sample was approximately 32, ranging from 19 to 58 years of age, with the majority being males (91 %) and married (72 %). Two-thirds of the sample were working on fixed morning, afternoon, or night shifts; one-third had 4–7 years of service, and another one-third had 12 years of service or more. Half of the sample are ‘high school graduates’ (without obtaining further educational certificates), while 22 % reported not finishing high school. The majority of the respondents had at least 4 or more years of working experience in the institution (91 %), with about 36 % reporting to work for 12 or more years. This indicates that most respondents are not new to police activities and have enough longevity in the profession. Also, most respondents were married (72 %) and hence could be subjected to work stress as well as high levels of social and family obligations.

**Table 2:** Demographic characteristics of respondents.

Variable	Observations	%
Age		
Mean	31.7 (7.3)	–
Min-Max	19–58	–
*Total*	*608*	*-*
**Gender**		
Male	604	91
Female	60	9
*Total*	*664*	*100*
**Marital status**		
Married	469	72
Never married	146	22
Previously married	38	6
*Total*	*653*	*100*
**Education**		
Not a high school graduate	148	22
High school graduate	334	50
Diploma/Bachelor	152	21
Post-graduate degree	29	4
*Total*	*663*	*100*
**Work experience**		
3 years of less	56	9
4–7 years	199	30
8–11 years	166	25
12 years or more	232	36
*Total*	*653*	*100*
**Work mode**		
Fixed shifts	436	66
Rotations	225	34
Total	*661*	*100*

#### 3.4.1 Dependent variable

##### 3.4.1.1 Sickness absence

Participants were asked about the number of days of sickness absence in the last 12 months. Response options were none, 1-3d, 4-7d, 8-28d, 29-90d and ≥91d. Many previous studies have used this self-reported sickness absence measure [[96], [97], [98]]. For this study, the last four responses in the sickness absence outcome were combined into one category (≥4d) as 74 % of reported absence ranged from 0 to 3 days, and the other categories combined to make the remaining observations.

#### 3.4.2 Independent variables

##### 3.4.2.1 Psychosocial work factors

These were 22 psychosocial items of the 49 items of the JCQ of Karasek's [99] model. Nine of these items were for job control, five were for job demand items, and eight were for workplace support items. These measures have been used previously to examine the risks of job control, demand, and support on sickness absence [33,97,100]. The questionnaire was translated into Arabic and administered for this study. The distribution of responses for the job control, demand, and support variables was used to classify participants into low, medium, and high categories. Participants were then arranged into four psychosocial work-status categories, namely, active (high control/high demand), passive (low control/low demand), relaxed (high control/low demand), and job strain (low control/high demand).

##### 3.4.2.2 Physical work exposures

The physical work items in the survey included questions regarding the intensity of nine physical exposures, with questions based on the European Working Conditions Survey [101]. For physical work exposures, participants were categorized into being either exposed less than half of the time or half of the time or more. A combined physical exposure measure was created for further analysis.

##### 3.4.2.3 Perceptions of organizational health and safety management systems

Using the UK Health and Safety Executive's guide for a successful health and safety management system [1], participants were asked seven questions regarding their perception of the health and safety management systems implemented by the Abu Dhabi Police. An item reflecting the overall perception of the health and safety management system was also created (from the aforementioned items, HSMST). Participants were categorized into having favourable (strongly agree and agree) and unfavourable (strongly disagree and disagree) perceptions.

##### 3.4.2.4 Other covariates

Other covariates included demographic variables, health indicators, lifestyle, social life factors, and other non-work factors**.**

##### 3.4.2.5 Survey reliability and validity

All the Cronbach's Alpha coefficients estimates for scales and subscales of the Arabic JCQ were above the recommended value of 0.70 [102]. This indicates the acceptability of internal consistency. As for construct validity testing, the results show that the psychological job demand scale and the two workplace support subscales have followed the theoretical framework. Most of the job control scale items were loaded on factor two, with one item only loading on factor three (CQ3) and two items (CQ4 & CQ5) loading on factors two and three. Finally, the intraclass correlation coefficient (ICC) estimates for the Arabic JCQ showed ‘fair’ reliability for the psychological job demand scale (ICC = 0.22) and moderate reliability for job control and workplace support (ICC = 0.54 and 0.50, respectively).

This study developed an Arabic version of the Job Content Questionnaire (the Arabic JCQ) and validated it linguistically and psychometrically using a sample from the Abu Dhabi Police. The linguistic validation was conducted in five main steps: forward translation, synthesis, back translation, expert review, and cognitive debriefing interviews. Changes were made to the Arabic JCQ after each step, and justification for changes was also documented. The psychometric validation was conducted using the survey sample with a 34.5:1 subject-to-variable ratio. The mean scores for psychological job demand, job control, and workplace support were 33.4, 65.5, and 24.7, respectively. These scores were generally comparable with the civilian sample of the US Quality of Employment Surveys [103].

### 3.5 Analysis

Descriptive analyses included the overall distribution of responses and missingness in each subcategory of work factor. The risk (odds) of having sickness absence of four days or more (≥4d) compared with 0–3 days (0-3d) were estimated using logistic regression. The association between each work and non-work factor and sickness absence categories was also examined. The risk of sickness absence was evaluated in relation to psychosocial work factors, employees' overall perception of the health and safety management system (HSMST), and the combined physical exposure variable (as this study primarily focuses on these factors) using logistic regression with those 0–3 days of sickness absence coded as ‘0’ and >4 days coded as ‘1’. While the hierarchical approach is becoming more popular for data that report repeated measures, in the case of nested data, and for unknown interactions [104], the characteristics of this data are better suited for the logistic regression model. Also, the binary coding of the outcome variable negated the use of other methods of analysis, such as the count models or the survival models. Count models could have suited well for the number of days the respondents reported the sickness absence. On the other hand, had the research inquiry analyzed the prediction of individuals taking four or more sickness days from 0 to 3 days [time to observed outcome], survival models would have been better suited for the analysis. However, the outcome is binary, and hence, the logistic regression model is more suitable for this analysis.

The analysis included one model with no other covariates (unadjusted model) and two models for the adjustment of covariates. This first stage of the analysis compared the unadjusted odds ratio estimates with those from Model I, which adjusted for age and gender, and Model II, which adjusted for other demographic factors associated with the outcome. These analyses were carried out using the restricted sample of responders with no missing values for any of the covariates in Model II.

The second stage of the analysis incorporated three additional models. Each included all the covariates in the preceding model. As with Model II, covariates were added only if they were also associated with the outcome. Model III additionally included social life, health, and lifestyle factors, while work setting factors were also added in Model IV. The fully adjusted model added the psychosocial and physical factors as well as employees’ perceptions of the health and safety management system. The analyses in this stage were conducted using the restricted sample of the fully adjusted model.

## 4 Results

Table 3 provides descriptive statistics regarding the distribution of work factors (determinants of interest) and their (unadjusted) association with sickness absence. This study evaluates only the following work factors: psychosocial work factors, overall perception of the health and safety management system, and combined physical exposure.

**Table 3:** Overall distribution of work factors and sickness absence.

						*Days of Sickness Absence (SA)*					
Work factor	n (%)			n with SA data		0d n (%)^2^		1-3d n (%)		4-7d n (%)	≥8d n (%)
**SICKNESS ABSENCE (Dep)** **PSYCHOSOCIAL FACTORS**				670		340 (51)		161 (24)	105 (16)		64 (10)
***Job control*** rowhead											
High	202 (30)			176		96 (55)	38 (22)		27 (15)		15 (9)
Medium	230 (34)			211		103 (49)	56 (27)		32 (15)		20 (9)
Low	252 (37)			231		113 (49)	57 (25)		38 (16)		23 (10)
*Total*	*684 (100)*			*618*		*312 (50)*	*151 (24)*		*97 (16)*		*58 (9)*
***Job demand*** rowhead											
Low	221 (31)			194		96 (49)	54 (28)		19 (10)		25 (13)
Medium	265 (37)			247		116 (50)	58 (23)		51 (21)		22 (9)
High	238 (33)			209		119 (57)	45 (22)		30 (14)		15 (7)
*Total*	*724 (100)*			*650*		*331 (51)*	*157 (24)*		*100 (15)*		*62 (10)*
***Co-workers support*** rowhead											
High	250 (35)			214		110 (51)	52 (24)		36 (17)		16 (7)
Medium	317 (44)			300		158 (53)	70 (23)		48 (16)		24 (8)
Low	153 (21)			137		61 (45)	37 (27)		16 (12)		23 (17)
*Total*	*720 (100)*			*651*		*329 (51)*	*159 (24)*		*100 (15)*		*63 (10)*
***Supervisors support*** rowhead											
High			281 (39)		244	119 (49)	64 (26)		40 (16)		21 (9)
Medium			284 (39)		268	141 (53)	58 (22)		46 (17)		23 (9)
Low			168 (23)		147	74 (50)	38 (26)		19 (13)		16 (11)
*Total*			*733 (100)*		*659*	*334 (51)*	*160 (24)*		*105 (16)*		*60 (9)*
***Workplace support*** rowhead											
High		265 (37)			231	122 (53)	56 (24)		36 (16)		17 (7)
Medium		295 (41)			276	136 (49)	66 (24)		49 (18)		25 (9)
Low		152 (21)			136	67 (49)	36 (26)		15 (11)		18 (13)
*Total*		*712 (100)*			*643*	*325 (51)*	*158 (25)*		*100 (16)*		*60 (9)*
***Psychological classification work***rowhead											
Relaxed		135 (20)			122	60 (49)	31 (25)		23 (19)		8 (7)
Passive		302 (45)			277	129 (47)	72 (26)		42 (15)		34 (12)
Active		117 (18)			98	58 (59)	20 (20)		12 (12)		8 (8)
Strain		114 (17)			105	57 (54)	24 (23)		17 (16)		7 (7)
*Total*		*668 (100)*			*602*	*304 (51)*	*147 (24)*		*94 (16)*		*57 (9)*
***HSE Management*** rowhead											
Favourable		561 (78)			509	254 (50)	120 (24)		89 (17)		46 (9)
Unfavourable		157 (22)			141	75 (53)	35 (25)		14 (10)		17 (12)
*Total*		*718 (100)*			*650*	*329 (51)*	*155 (24)*		*103 (16)*		*63 (10)*
***Combined Physical Exposure*** rowhead											
Low		233 (37)			220	102 (46)	49 (22)		42 (19)		27 (12)
Moderate		188 (30)			170	98 (58)	40 (24)		19 (11)		13 (8)
High		215 (34)			192	97 (51)	48 (25)		29 (15)		18 (9)
*Total*		*636 (100)*			*582*	*297 (51)*	*137 (24)*		*90 (15)*		*58 (10)*

Parenthesis presents the percentages.

### 4.1 Descriptive statistics with unadjusted model

The psychological work status of 45 % of the participants is classified as ‘passive’ while 17 % were considered as ‘strain’ (Table 3). More than two-thirds of employees reported an overall favourable perception of the health and safety management system. In this police sample 51 % had no days while 26 % and 10 % had ≥ 4d and ≥ 8d of sickness absence respectively. The highest category of response for all work factors was medium, with over 3/4th of all responses in the top two categories.

### 4.2 Adjustment models

This study evaluated the influence of psychosocial work factors, physical work factors and employees’ perception of organizational health and safety management system on the risk of sickness absence. Table 4 shows the odds ratio of the association between work factors and the risk of sickness absence in three models: unadjusted, adjusting for age and gender (model I), and adjusting additionally for other demographic factors (partner working status as a covariate (model II).

**Table 4:** Work factors and the odds ratio of sickness absence of three statistical models^a^.

Work factor	Unadjusted	Model I	Model II
*Job control (n = 458)*			
High	*Reference*		
Medium	1.03 (0.60–1.76)	1.11 (0.64–1.91)	1.09 (0.63–1.88)
Low	1.07 (0.63–1.81)	1.05 (0.61–1.79)	1.07 (0.63–1.84)
***Job demand (n = 477)***			
Low	*Reference*		
Medium	1.26 (0.75–2.12)	1.30 (0.77–2.21)	1.28 (0.75–2.18)
High	0.93 (0.53–1.60)	1.00 (0.57–1.75)	1.00 (0.58–1.77)
***Psychological work classification (n = 449)***			
Relaxed	*Reference*		
Passive	1.21 (0.61–2.11)	1.28 (0.72–2.28)	1.30 (0.73–2.30)
Active	0.89 (0.43–1.81)	0.99 (0.48–2.04)	1.03 (0.49–2.14)
Strain	0.88 (0.44–1.76)	0.99 (0.49–2.00)	1.02 (0.50–2.06)
***Co-workers support (n = 476)***			
High	*Reference*		
Medium	1.02 (0.63–1.64)	1.03 (0.64–1.68)	1.03 (0.63–1.67)
Low	1.25 (0.70–2.22)	1.24 (0.69–2.24)	1.26 (0.70–2.28)
***Supervisors support (n = 481)***			
High	*Reference*		
Medium	1.05 (0.66–1.66)	1.05 (0.66–1.68)	1.06 (0.66–1.69)
Low	0.95 (0.53–1.68)	0.88 (0.49–1.59)	0.92 (0.51–1.65)
***Workplace support (n = 471)***			
High	*Reference*		
Medium	1.19 (0.75–1.91)	1.24 (0.77–2.00)	1.22 (0.75–1.98)
Low	1.17 (0.65–2.10)	1.12 (0.61–2.04)	1.17 (0.64–2.14)
***Overall perception of HSE management system (n = 475) (HSMST)***			
Favourable	*Reference*		
Unfavourable	0.80 (0.47–1.35)	0.80 (0.47–1.36)	0.84 (0.49–1.44)
***Combined physical exposure (n = 428)***			
Low	*Reference*		
Moderate	**0.44 (0.25–0.77)**	**0.45 (0.25–0.80)**	**0.45 (0.25–0.80)**
High	**0.53 (0.31–0.89)**	**0.50 (0.29–0.86)**	**0.53 (0.30–0.92)**

Model I: Adjusted for age and gender Model II: Previous model + partner working.

a Using the restricted sample of model II with no missing covariate data.

In general, the associations between work factors and the odds of sickness absence in the unadjusted model (Table 4) of this restricted sample are similar to the odds ratio of the unadjusted model of the full sample (Table 3). The only difference is the significant protective effect in officers with high combined physical exposures (OR = 0.53, 95%CI = 0.31–0.89). Little change in the associations was seen between models I and II.

Using the restricted sample of model II, exposure to five physical work factors (vibration, noise, working in painful positions, working while standing, and repetitive arm/hand movements) half of the working time or more was associated with lower odds ratios of sickness absence than seen from the unadjusted model, ranging from OR = 0.43 (95%CI = 0.26–0.71) for exposure to noise to OR = 0.64 (95%CI = 0.41–0.98) for exposure to vibration.

The second stage of adjustments was carried out using six statistical models (Table 5). The influence of work factors on the odds of sickness absence in this restricted sample in the unadjusted model and model I was generally similar to that of the full police sample. The influence of work factors on the odds of sickness absence in the unadjusted model and model I in the full sample and the restricted sample of the fully adjusted model Is shown in Table 5. The only major difference is the significant protective effect seen in employees with high (versus low) exposure to the combined physical work exposures (OR = 0.38, 95%CI = 0.21–0.70). This association became non-significant in the fully adjusted model.

**Table 5:** Work factors and the odds ratio of sickness absence of six statistical models.^a^

Work factor	Unadjusted	Model I	Model II	Model III	Model IV	Fully adjusted
Job control (n = 335)						
High	*Reference*					
Medium	1.05 (0.56–1.96)	1.13 (0.60–2.14)	1.09 (0.57–2.06)	1.28 (0.64–2.55)	1.23 (0.62–2.46)	1.08 (0.52–2.26)
Low	1.23 (0.67–2.24	1.22 (0.66–2.25)	1.25 (0.67–2.31)	1.27 (0.66–2.47)	1.26 (0.65–2.44)	1.29 (0.64–2.58)
***Job demand (n = 348)***						
Low	*Reference*					
Medium	1.17 (0.63–2.16)	1.22 (0.66–2.28)	1.16 (0.62–2.18)	1.38 (0.70–2.72)	1.50 (0.75–2.99)	1.46 (0.71–2.99)
High	0.90 (0.47–1.69)	0.99 (0.52–1.88)	0.99 (0.52–1.90)	1.07 (0.53–1.90)	1.14 (0.56–2.32)	1.20 (0.57–2.52)
***Psychological work classification (n = 330)***						
Relaxed	*Reference*					
Passive	1.40 (0.73–2.67)	1.48 (0.77–2.87)	1.04 (0.77–2.88)	1.71 (0.84–3.50)	1.78 (0.86–3.67)	1.79 (0.85–3.77)
Active	0.90 (0.40–2.05)	1.01 (0.43–2.33)	1.07 (0.46–2.49)	1.05 (0.43–2.54)	1.10 (0.45–2.67)	1.23 (0.48–3.14)
Strain	1.02 (0.47–2.19)	1.13 (0.51–2.47)	1.16 (0.53–2.55)	1.12 (0.48–2.57)	1.17 (0.50–2.71)	1.15 (0.48–2.75)
***Co-workers support (n = 348)***						
High	*Reference*					
Medium	0.89 (0.52–1.53)	0.89 (0.51–1.55)	0.86 (0.49–1.56)	0.86 (0.48–1.54)	0.86 (0.48–1.55)	0.88 (0.48–1.63)
Low	1.15 (0.59–2.23)	1.12 (0.57–2.20)	1.11 (0.56–2.19)	0.93 (0.45–1.93)	0.96 (0.46–2.00)	1.19 (0.55–2.57)
***Supervisors support (n = 348)***						
High	*Reference*					
Medium	1.12 (0.66–1.90)	1.15 (0.67–1.97)	1.11 (0.64–1.91)	1.27 (0.72–2.25)	1.31 (0.74–2.33)	0.79 (0.72–2.37)
Low	0.86 (0.45–1.66)	0.82 (0.42–1.61)	0.84 (0.43–1.64)	0.68 (0.32–1.42)	0.73 (0.34–1.54)	0.79 (0.37–1.71)
***Workplace support (n = 344)***						
High	*Reference*					
Medium	1.13 (0.66–1.92)	1.17 (0.68–2.01)	1.11 (0.64–1.93)	1.23 (0.69–2.21)	1.25 (0.70–2.24)	1.28 (0.70–2.34)
Low	1.10 (0.51–1.98)	0.96 (0.47–1.91)	0.99 (0.50–1.97)	0.82 (0.39–1.74)	0.84 (0.39–1.79)	1.00 (0.45–2.18)
***Overall perception of HSE management (n = 349)***						
Favourable	*Reference*					
Unfavourable	0.88 (0.49–1.57)	0.87 (0.48–1.57)	0.94 (0.51–1.72)	0.91 (0.48–1.71)	0.98 (0.52–1.85)	1.16 (0.60–2.20)
***Combined physical exposure (n = 344)***						
Low	*Reference*					
Moderate	**0.45 (0.24–0.82)**	**0.46 (0.25–0.85)**	**0.46 (0.25–0.86)**	**0.42 (0.22–0.81)**	**0.43 (0.22–0.83)**	**0.43 (0.22–0.83)**
High	**0.38 (0.21–0.70)**	**0.37 (0.20–0.68)**	**0.38 (0.20–0.72)**	**0.33 (0.17–0.64)**	**0.33 (0.17–0.65)**	**0.33 (0.17–0.65)**

Model I: Adjusted for age and gender Model II: Previous model + partner working status Model III: Previous model + private life support, frequency of meeting friends, tragic events last 12 months, self-rated health, work ability, smoking shisha and presenteeism. Model IV: Previous model + dominant work gender Fully adjusted: previous model + noise, carrying heavy objects, standing and repetitive movement.

a Using the restricted sample of the fully adjusted model with no missing covariate data.

In general, there was little change in the associations between the six models for job control, co-workers’ and workplace support, and participants in the job ‘strain’ category compared with those in the ‘relaxed’ category. A marked increase in the odds of sickness absence, non-significant still, was noticed in model III for those in the ‘passive’ job category (versus ‘relaxed’) (67 %), medium (versus low) job demand (22 %) and medium (versus high) supervisors' support (16 %).

In summary, psychosocial work factors and perceptions of health and safety management systems were not significant predictors of sickness absence in this police sample. Surprisingly, officers who reported high combined physical exposures (compared with those reporting low) had a significant protective effect from sickness absence after adjusting for all covariates.

## 5 Discussion

This study aimed to evaluate the influence of work factors on self-reported sickness absence using the Job-Control model. The risk of sickness absence in this study refers to the risk of reporting ≥4d compared with 0-3d of sickness absence. In general, the study found no association between psychosocial work factors (job control, demand, and workplace support) and employees’ perception of health and safety management with the risk of sickness absence. This was seen both with and without adjusting for age, gender, and other work and non-work covariates. A significant decrease in risk of sickness absence was seen in officers reporting moderate or high (compared with low) combined physical exposures after adjusting for all covariates.

### 5.1 Psychosocial work factors and the risk of sickness absence

This study found no association between job control and the risk of sickness absence, in contrast to findings from other studies. Niedhammer et al. [98] and Lesuffleur et al. [105] found low job control to result in a significant increase in the risk of sickness absence, respectively, for males and for females. Other studies have supported this inverse relationship [27,33,106].

There was also no association between psychological job demand and the risk of sickness absence in this study. Previous studies which evaluated this relationship showed mixed results. An increase in the risk of sickness absence with high job demand by 21 % (74) and 34 % for males and 48 % for females (96) have been reported, which was also supported by other scholars [27,82,105]. In the British Whitehall Study, male employees with high job demand had a protective effect from short (OR = 0.75, 0.69–0.80) and long-term (OR = 0.76, 0.64–0.90) sickness absence [29] while other studies showed a non-significant association [71,73,74,107] reported an overall increase of 21 % in risk of sickness.

absence for high demand jobs. Other studies reported an increase in both male and female employees [27,31,82,97,105]. However, other studies report a non-significant association with sickness absence, irrespective of gender or short or long-term sickness absence.

Officers who fit the ‘job strain’ category did not have a significant increase in the risk of sickness absence in this study. This contradicts the majority of previous studies that showed increases in the risks of the frequency and the duration of sickness absenteeism [28,33,[108], [109], [110]]. In their meta-analysis, Duijts et al. [111] found that job strain results in a significant 20 % and 48 % increase in both short-term (3 days or less) and long-term sickness absence, respectively.

This study also found no association between support at work and the risk of sickness absence, which concurs with some studies [27,60,75] and disagrees with other studies that showed that support is a significant predictor of sickness absences (high support reduced sickness absence) [31,72,73,112].

Literature on the influence of work factors on the risk of sickness absence in the police force is limited [36,113]. It is to be noted that only one study [38] investigated the impact of psychosocial work factors on sickness absence in the police force. This Italian study also found non-significant associations between job control, job demand, and workplace support and the risk of sickness absence. Thus, the lack of association could be attributed to factors relating to the policing job itself. For instance, research has shown that when compared with psychosocial and physical work stressors, administrative/professional pressures are the most frequent work stressors reported by police officers. Police stress resulting from administrative/professional pressures is not only the result of low job control but can also be captured by the criticism of the police by the public, the imbalance between effort and reward, and work and family conflicts [113,114]. In addition, previous studies have also shown that police officers are more resilient to stress than civilians [[115], [116], [117]], which could explain the lack of association between psychological job factors and sickness absence.

The male-dominated hierarchal semi-military structure of law enforcement agencies is an inherent culture that makes it difficult for police employees to admit the existence of problems and discourages officers from seeking help. In the current study, only 9 % of police employees were female. Thus, investigating the association between psychological work factors and the risk of sickness absence in males and females separately was not feasible. Gender stratified analyses could have provided reasons for the lack of association between psychosocial work factors and sickness absence [31,50,74].

Similar cultural influences on the workforce can be observed in the Gulf region such as Saudi Arabia, Qatar, and Kuwait. In this organizational structure, police officers are trained to withstand stress by controlling emotions and solving their problems and that of others while on duty [118,119]. Finally, investigating stress (job strain in the current study) in police employees is generally difficult as they may be afraid that they can be identified as individuals with stress, which may result in termination from work or re-allocation [120].

The differences in findings with respect to the influence of psychological work factors and risk of sickness absence between the current study and previous studies could also be attributed to differences in the use of sickness absence measures or how the risk of sickness absence was measured [33,50,121] the stratification of results by socio-demographic factors such as gender [42,50] and socioeconomic status [28]. For instance, Roelen et al. [121] found that high psychological job demand increases the risk of long-term sickness absence (>7 days) but not shorter periods of sickness absence. Kivimaki et al. [27] also found similar non-significant associations among employees in the public sector. This could reflect other complex organizational processes factors not covered in this study, such as social network and organizational norms [122]. Finally, sickness absence could have been influenced by sources of support for police employees other than those examined in this study, such as non-police friends, departmental support, and support from the public [114,123]. Future studies in the region and in the UAE can provide more insights into this relationship.

### 5.2 Overall perception of health and safety management system and physical work exposures

It can be argued that employees' perception of the health and safety management system may influence sickness absence indirectly. Holding unfavourable perceptions of the health and safety management system may reduce employees’ job satisfaction, which has been shown to increase the risk of sickness absence [51,124]. In the current study, however, officers with an unfavourable overall perception of the health and safety management system had a non-significant protective effect from sickness absence which remained non-significant but with an increase in the risk after adjusting for all covariates.

The reasons behind this lack of association are unclear, and comparison with other studies is difficult due to the lack of studies on this relationship. It can be argued that employees could be highly satisfied with certain aspects of the job (such as salary), which may reduce the influence of holding an unfavourable perception of the health and safety management system on the risk of sickness absence.

In this study, officers reporting high combined physical exposures had a significant protective effect from sickness absence after adjusting for all covariates. Adverse physical working conditions could cause occupational stress [77], which, in turn, may increase sickness absenteeism [28,78]. However, it is unclear why the relationship was significant in the unexpected direction. Adverse physical working conditions may reduce one's job satisfaction [51], which has been shown to increase sickness absence [125,126]. Thus, it can be argued that police employees reporting unfavourable working conditions might have still been satisfied with their job and hence, had a protective effect from sickness absence.

The unexpected relationship could also result from employees' overstating or exaggeration of their exposure to physical work hazards, a problem also noted by Bockerman & Ilmakunnas [51]. In their analysis of predictors of sickness absence, Allebeck and Mastekaasa [76] stated that evidence on the influence of physical working conditions on sickness absence is ‘limited’ and that studies were not consistent in their use of self-reported measures of the physical work environment. Therefore, it is difficult to compare the findings of the current study with previous literature.

## 6 Conclusion and implications

This research evaluated predictors of sickness absence, particularly work factors, including psychosocial work factors, physical work exposure factors, and employee's perception of the health and safety management system in the Abu Dhabi Police. Previous studies have indicated that the validity of the self-reported sickness absence measure compared with electronically registered sickness absence data is generally good [74,127].

This research found that among work factors, only the combined physical work measure (incorporating nine factors) predicted sickness absence in the police force. In this research, it was hypothesized that psychosocial, physical, and employee perceptions of health and safety management systems predict sickness absence.

### 6.1 Socio-cultural context

The policing job is generally male-dominated, hierarchal, and with a semi-military rigid structure. For example, Abu Dhabi Police use a similar ranking for police officers and a rigid chain of command and reporting systems as that adopted in the military. Therefore, officers may already anticipate certain unfavourable job characteristics such as low job control, high job demand and job strain in their job [128]. Given that officers also have been reported to have high levels of readiness to deal with stress at work, control their emotions, and ‘solve problems’ rather than report ‘having problems’ [119], high job demand and low control at work may not necessarily increase the risk of sickness absence in the police.

Also, the Abu Dhabi police is primarily composed of UAE nationals, which could have led to lesser variability in the psycho-social factors. Also, the national working population, male and female combined, is less than 11 % of the total non-national working population; the inclusion of the expat population in the study could bring more insights into the relation of the independent variables predicting sickness absence in Abu Dhabi police. With the leadership of UAE increasing the Emiratization of both the private and public organizations within the UAE, unemployment among nationals is estimated to reduce significantly over time. This can increase the national workforce with higher female representation in the study for the future.

In addition, in the Abu Dhabi Police, all sickness absence, regardless of spell duration, has to be reported and registered in both the electronic human resource file of the employee and the medical file of the employee in the medical service department. This rigid system is an essential method for monitoring the occupational health of officers. Nevertheless, when officers encounter work or non-work problems that may necessitate taking sick leave, they may feel reluctant to admit problems at work and avoid taking sickness absence as this may indicate weakness. More frequent sickness absences could subject them to re-allocation to less stressful jobs, which may, in turn, have negative outcomes (reduced salary and less attractive jobs) [120].

This inherent policing culture that discourages reporting psychological work-related problems to colleagues or supervisors may result in shifting the potential consequences of these problems outside of work. For example, high rates of domestic violence were seen in police officers [129]. Previous studies have also indicated that employees often tend to share work concerns with friends and family more than utilizing other forms of support provided at work [119,130]. It may be that this help-seeking outside of the work environment is exacerbated in police officers, which should be investigated in future studies.

Thus, when faced with work problems, officers tend to seek support from outside of work and possibly avoid taking sick leave as a mechanism to cope with stress. This could explain the lack of association between workplace support and sickness absence in the police. Social support at work has a buffering influence on stress, ‘the stress-buffering hypothesis’ protecting workers from the pathological consequences of stressful experiences [[131], [132], [133], [134]].

### 6.2 Perception of health management system

The reasons for the lack of association between employees' overall perception of the health and safety management system and the risk of sickness absence and the protective effect of adverse physical working conditions on the risk of sickness absence are not clear. Previous studies have shown that holding favourable occupational health and safety perceptions promotes safe working [135], minimises occupational injuries (leading indirectly to sickness absence) [136] and increases employees’ satisfaction with work [137]. Therefore, it may be that officers could be highly satisfied with other aspects of policing work that minimise the potential impact of holding negative perceptions of the health and safety management system on the risk of sickness absence.

In a Norwegian study, Høivik et al. [138] also found that employees' perceptions of health and safety management systems did not predict sickness absence or occupational injuries. The sample of the latter study was 10,908 employees working in the Petroleum industry and were primarily exposed to chemical hazards. The study was similar to the current study in that 90 % of the sample were males. Their study also found a significant negative correlation between confidence in management and style of leadership and sickness absence. Thus, the absence of association between employees' perceptions of the health and safety management systems and the risk of sickness absence in the current study could be attributed to employees’ higher confidence in police management. Further studies are needed to examine the potential moderation effect of having confidence in leadership on the relationship between holding unfavourable perceptions of the health and safety management system and the risk of sickness absence.

For instance, the use of self-reported measures of working conditions, in particular when evaluating physical work factors, is problematic [76] and is prone to exaggeration by employees [51]. This could be resolved by using an objective measure [69,139], which will provide more insights into the true relationship between physical work factors and sickness absence in the police.

As for work predictors of sickness absence, the findings of this research do not support the idea that unfavourable psychosocial work factors increase the risk of sickness absence in the police. There was only one study conducted by Magnavita and Garbarino [38] evaluating the influence of psychosocial work factors on sickness absence in the police force. The latter study also showed no association between psychosocial work factors and sickness absence.

Notably, the current study is the first to evaluate the influence of employees’ perception of health and safety management systems and physical working conditions on the risk of sickness absence in the police. The research found that unfavourable perceptions of health and safety management systems did not predict sickness absence which is similar to the findings of another study carried out by Høivik et al. [138] using a sample from the Norwegian Petroleum industry. This research, however, adds new knowledge to existing literature that adverse physical work factors have protective effects from sickness absence in the police.

### 6.3 Implications for future research

In the future, gaining further insights into work predictors of sickness absence in the police force necessitates focusing on five themes. First, obtaining data regarding other crucial organisational aspects will help deepen the understanding of the association between work predictors and the risk of sickness absence. These include satisfaction with various job factors [139,140]; participation and involvement in decision making [141]; information sharing [142] and the extent to which the organisation values employees’ contribution [143].

Secondly, evaluating other factors that are specific to the policing job, such as sleep disorders [144], divisional level exposures [145], and stress coping styles [146], is also essential. For example, Habersaat et al. [146] showed a significant difference in the reporting of negative health outcomes in officers working in different police divisions, which was associated with differences in occupational exposures.

Thirdly, the validity of future research findings can be improved by asking external examiners to assess work predictors, particularly physical work factors [69,138]. This will minimise issues associated with subjective measures [76], such as employees’ tendency to exaggerate levels of work exposure [51]. This will introduce another subjective bias (extent of the accuracy of the external assessor(s)); thus, the validity is improved by the selection of experienced examiners and ensuring that any support, training, and resources needed are provided in order for the examiner to conduct assessment appropriately.

Fourthly, when analysing results of future research, stratifying results by gender, age or job type will provide more in-depth explanations for differences in results. One of the features of policing jobs is the gender segregation of tasks, as male officers are more likely to work in hazardous jobs while female officers predominantly work with women or children and sexually abused victims [147,148]. In addition, help-seeking behaviour differs between males and females. For example, Berg et al. [119] found that female police officers seek help from health professionals more often than male officers. In the current research, gender-stratified analyses could have provided reasons for the lack of association between psychosocial work factors and sickness absence [31,50].

Finally, 22 % of police employees who agreed to participate in this research (146/760) also provided their ID and agreed to participate in any future studies as well as consenting for their data to be linked with other data in the future. Thus, the data obtained from this survey can be linked (using the employees' ID) with future data collected using surveys or from a register, converting the cross-sectional data into a longitudinal study to give insights into various outcomes. For example, baseline work factors could be linked with employees’ performance, productivity, visits to health care professionals, opinions regarding organisational specific outcomes, future sickness absence, disability retirement, turnover and early retirement intention.

### 6.4 Limitations of the study

There are four main limitations of this study. This study used self-reported measures, which are subject to recall bias as in other similar studies [40,119,149]. The cross-sectional design of the study makes it difficult to establish causal inference: if the exposure to work factors led to the outcome or if the relationship existed in the opposite direction [150]. Missing data was another limitation of this study. It resulted in reducing the sample size to almost half when adjusting for various covariate groups. Finally, females were under-represented in this sample of police officers which made it difficult to carry out stratified analyses of results. These inherited cultural characteristics can be observed in most Middle Eastern countries, especially the Gulf Cooperative Countries. Henceforth, the implications of having a higher percentage of females in the police can provide more insights and higher replicability to this study.

Barring these limitations, the strength of this study is that it is one of the first to evaluate the influence of employees’ perception of organizational health and safety management system on the risk of sickness absence. This study was carried out in one of the largest organization in the Emirate of Abu Dhabi, the findings of the study could be compared with future occupational health research in the other emirates of Abu Dhabi and the Middle Eastern region. The limitation of this study emphasizes the opportunity to build on the socio-cultural setup of the police departments in the region. For researchers interested in cross-cultural studies, this study introduces the organizational approaches, socio-demographic dynamics, and process to collect and examine future research inquiries within the police department in Abu Dhabi and, to a good extent, for the Middle Eastern region, given the similar cultural and social fabric. One such research inquiry is the need to examine the potential moderation effect of having confidence in leadership on the relationship between holding an unfavourable perception of the health and safety management system and the risk of sickness absence.

Statement of Ethical Consideration: All subjects gave their informed consent for inclusion before they participated in the study. The study was conducted in accordance with the Declaration of Helsinki, and the protocol was approved by the Ministry of Interior, Abu Dhabi Police (No. 2000-33/11/5481)

## Data availability statement

The data that support the findings of this study are not available due to privacy restrictions on data sharing by the original entity [AD Police] and adhering to privacy restrictions regarding the information about employees of the Abu Dhabi Police. Henceforth, the authors do not have permission to share the data of this study.

Declaration of competing interest: The authors declare that they have no known competing financial interests or personal relationships that could have appeared to influence the work reported in this paper.

## CRediT authorship contribution statement

**Faisal Almurbahani Alkaabi:** Supervision, Methodology, Funding acquisition, Formal analysis, Data curation, Conceptualization. **Praveen Kumar Maghelal:** Writing – review & editing, Writing – original draft, Supervision, Project administration, Methodology, Formal analysis. **Jana Ahmed AlShkeili:** Resources, Investigation, Data curation.

## Declaration of competing interest

The authors declare that they have no known competing financial interests or personal relationships that could have appeared to influence the work reported in this paper.
